# Supplementary material for: Medical care needs for patients receiving home healthcare in Taiwan: Do gender and income matter?
Source: PLoS One. 2021 Feb 25;16(2):e0247622. doi: 10.1371/journal.pone.0247622 (PMC7906386; doi:10.1371/journal.pone.0247622)

PLOS ONE

Medical care needs for patients receiving home healthcare in Taiwan: Do gender and income matter?

--Manuscript Draft--

**Manuscript Number:**	PONE-D-20-32788
**Article Type:**	Research Article
**Full Title:**	Medical care needs for patients receiving home healthcare in Taiwan: Do gender and income matter?
**Short Title:**	The effects of gender and income on medical care needs
**Corresponding Author:**	Ping-Jen Chen<br>University College London<br>London, UNITED KINGDOM
**Keywords:**	Medical Care Needs; Home Healthcare; Gender; Feminization of Poverty
**Abstract:**	Studies about medical care needs, represented by the resource utilization group (RUG), for home healthcare (HHC), previously focused on disease patterns but not gender and income differences. We used the Taiwan National Health Research Insurance Database from 1997 to 2013 to examine trends in medical care needs for patients who received HHC, and why there is a gender gap in medical care needs. We wanted to clarify three questions: 1. Are females at a higher level of medical care needs for HHC than males, 2. Does income relate to medical care needs and how is this different for females and males? 3. Is the interaction term (gender and income) related to the likelihood of medical care needs? In addition, we analyzed the pathways to gender disparity in medical care need by using chi-square tests and multiple-ordinal regression models. Results showed that women in the lowest level of income have higher medical care needs than men. Income moderated the effect of gender on the likelihood of higher medical care needs. The results are consistent with the perspective of fundamental causes of disease and feminization of poverty theory. In conclusion, the secular trend of levels of RUG in patients receiving HHC is growing but the frequency of the most severe levels of RUG is declining. To reduce the double burden of female poverty at the higher levels of RUG in patients who received HHC, policymakers should provide financial support for older women below the threshold of the poverty line.
**Order of Authors:**	Fang-Yi Huang<br>Chung-Han Ho<br>Jung-Yu Liao<br>Sang-Ju Yu<br>Kai-Ping Zhang<br>Ping-Jen Chen
**Additional Information:**	
**Question**	**Response**
**Financial Disclosure**<br><br>Enter a financial disclosure statement that describes the sources of funding for the work included in this submission. Review the submission guidelines for detailed requirements. View published research articles from *PLOS ONE* for specific examples.	The author(s) received no specific funding for this work.

Powered by Editorial Manager® and ProduXion Manager® from Aries Systems Corporation

This statement is required for submission and **will appear in the published article** if the submission is accepted. Please make sure it is accurate.<br><br>**Unfunded studies**<br>Enter: *The author(s) received no specific funding for this work.*<br><br>**Funded studies**<br>Enter a statement with the following details:<br>• Initials of the authors who received each award<br>• Grant numbers awarded to each author<br>• The full name of each funder<br>• URL of each funder website<br>• Did the sponsors or funders play any role in the study design, data collection and analysis, decision to publish, or preparation of the manuscript?<br>• **NO** - Include this sentence at the end of your statement: *The funders had no role in study design, data collection and analysis, decision to publish, or preparation of the manuscript.*<br>• **YES** - Specify the role(s) played.<br><br>* typeset	
**Competing Interests**<br><br>Use the instructions below to enter a competing interest statement for this submission. On behalf of all authors, disclose any competing interests that could be perceived to bias this work—acknowledging all financial support and any other relevant financial or non-financial competing interests.<br><br>This statement **will appear in the published article** if the submission is accepted. Please make sure it is accurate. View published research articles from *PLOS ONE* for specific examples.	The authors have declared that no competing interests exist.

**NO authors have competing interests**<br><br>Enter: *The authors have declared that no competing interests exist.*<br><br>**Authors with competing interests**<br><br>Enter competing interest details beginning with this statement:<br><br>*I have read the journal's policy and the authors of this manuscript have the following competing interests: [insert competing interests here]*<br><br>* typeset	
**Ethics Statement**<br><br>Enter an ethics statement for this submission. This statement is required if the study involved:<br><br>• Human participants<br>• Human specimens or tissue<br>• Vertebrate animals or cephalopods<br>• Vertebrate embryos or tissues<br>• Field research<br><br>Write "N/A" if the submission does not require an ethics statement.<br><br>General guidance is provided below. Consult the submission guidelines for detailed instructions. **Make sure that all information entered here is included in the Methods section of the manuscript.**	The study has been granted exemption from review by the Institutional Review Board of Chi Mei Medical Center (IRB No. 10410-E01).

**Format for specific study types**<br><br>**Human Subject Research (involving human participants and/or tissue)**<br>• Give the name of the institutional review board or ethics committee that approved the study<br>• Include the approval number and/or a statement indicating approval of this research<br>• Indicate the form of consent obtained (written/oral) or the reason that consent was not obtained (e.g. the data were analyzed anonymously)<br><br>**Animal Research (involving vertebrate animals, embryos or tissues)**<br>• Provide the name of the Institutional Animal Care and Use Committee (IACUC) or other relevant ethics board that reviewed the study protocol, and indicate whether they approved this research or granted a formal waiver of ethical approval<br>• Include an approval number if one was obtained<br>• If the study involved *non-human primates*, add *additional details* about animal welfare and steps taken to ameliorate suffering<br>• If anesthesia, euthanasia, or any kind of animal sacrifice is part of the study, include briefly which substances and/or methods were applied<br><br>**Field Research**<br><br>Include the following details if this study involves the collection of plant, animal, or other materials from a natural setting:<br>• Field permit number<br>• Name of the institution or relevant body that granted permission	
**Data Availability**<br><br>Authors are required to make all data underlying the findings described fully available, without restriction, and from the time of publication. PLOS allows rare exceptions to address legal and ethical concerns. See the PLOS Data Policy and FAQ for detailed information.	No - some restrictions will apply

Powered by Editorial Manager® and ProduXion Manager® from Aries Systems Corporation

A Data Availability Statement describing where the data can be found is required at submission. Your answers to this question constitute the Data Availability Statement and **will be published in the article**, if accepted.<br><br>**Important:** Stating 'data available on request from the author' is not sufficient. If your data are only available upon request, select 'No' for the first question and explain your exceptional situation in the text box.<br><br>Do the authors confirm that all data underlying the findings described in their manuscript are fully available without restriction?	
**Describe where the data may be found in full sentences. If you are copying our sample text, replace any instances of XXX with the appropriate details.**<br><br>• If the data are **held or will be held in a public repository**, include URLs, accession numbers or DOIs. If this information will only be available after acceptance, indicate this by ticking the box below. For example: *All XXX files are available from the XXX database (accession number(s) XXX, XXX.*).<br>• If the data are all contained **within the manuscript and/or Supporting Information files**, enter the following: *All relevant data are within the manuscript and its Supporting Information files.*<br>• If neither of these applies but you are able to provide **details of access elsewhere**, with or without limitations, please do so. For example:<br><br>*Data cannot be shared publicly because of [XXX]. Data are available from the XXX Institutional Data Access / Ethics Committee (contact via XXX) for researchers who meet the criteria for access to confidential data.*<br><br>*The data underlying the results presented in the study are available from (include the name of the third party*	The data underlying this study are from the National Health Insurance Research Database (NHIRD), which has been transferred to the Health and Welfare Data Science Center (HWDC). Interested researchers can obtain the data through formal application to the HWDC, Department of Statistics, Ministry of Health and Welfare, Taiwan (http://dep.mohw.gov.tw/DOS/np-2497-113.html). All applications are reviewed for approval of data release and applicants must follow the Computer-Processed Personal Data Protection Law (http://www.winklerpartners.com/?p=987) and related regulations of National Health Insurance Administration.

and contact information or URL).<br>• This text is appropriate if the data are owned by a third party and authors do not have permission to share the data.<br><br>* typeset	
Additional data availability information:	

Manuscript
Click here to access/download;Manuscript;Manuscript_v6_HHC-Gender-

Full title: Medical care needs for patients receiving home healthcare in Taiwan: do gender and income matter?

Short title: The effects of gender and income on medical care needs

Author:

Fang-Yi Huang[1], Chung-Han Ho[2,3], Jung-Yu Liao[4], Sang-Ju Yu[5], Kai-Ping Zhang[5], Ping-Jen Chen[6,7,8,*]

[1] Institute of European and American Studies, Academia Sinica, Taipei, Taiwan
[2] Department of Medical Research, Chi-Mei Medical Center, Tainan, Taiwan
[3] Department of Hospital and Health Care Administration, Chia Nan University of Pharmacy and Science, Tainan, Taiwan
[4] Institute of Population Health Sciences, National Health Research Institutes, Miaoli, Taiwan
[5] Home Clinic Dulan, Taitung and Taipei, Taiwan
[6] Marie Curie Palliative Care Research Department, Division of Psychiatry, University College London, London, United Kingdom
[7] Department of Family Medicine and Division of Geriatrics and Gerontology, Kaohsiung Medical University Hospital, Kaohsiung Medical University, Kaohsiung, Taiwan
[8] School of Medicine, Kaohsiung Medical University, Kaohsiung, Taiwan
[*] Corresponding author
Email: pingjen.chen@gmail.com (PJC)

Abstract

Studies about medical care needs, represented by the resource utilization group (RUG), for home healthcare (HHC), previously focused on disease patterns but not gender and income differences. We used the Taiwan National Health Research Insurance Database from 1997 to 2013 to examine trends in medical care needs for patients who received HHC, and why there is a gender gap in medical care needs. We wanted to clarify three questions: 1. Are females at a higher level of medical care needs for HHC than males, 2. Does income relate to medical care needs and how is this different for females and males? 3. Is the interaction term (gender and income) related to the likelihood of medical care needs? In addition, we analyzed the pathways to gender disparity in medical care need by using chi-square tests and multiple-ordinal regression models. Results showed that women in the lowest level of income have higher medical care needs than men. Income moderated the effect of gender on the likelihood of higher medical care needs. The results are consistent with the perspective of fundamental causes of disease and feminization of poverty theory. In conclusion, the secular trend of levels of RUG in patients receiving HHC is growing but the frequency of the most severe levels of RUG is declining. To reduce the double burden of female poverty at the higher levels of RUG in patients who received HHC, policymakers should provide financial support for older women below the threshold of the poverty line.

Keywords: Medical Care Needs, Home Healthcare, Gender, Feminization of Poverty, Taiwan

Introduction

Home healthcare and medical care needs

The needs of home healthcare (HHC) is increasing due to the aim of aging in place in the era of global population aging. Compared to a larger literature on the effects of social determinants on medical care needs [1–5], however, fewer have concentrated on HHC patients who live at home. Some studies investigated the association between the use of home health services, the access of device use [5], the number of readmission [6], and duration of rehabilitation care [7]. Little was known about specialized medical care needs such as tracheostomy and urinal catheterization care, intravenous injection, or colostomy irrigation in the HHC recipients.

Gender and medical care needs for HHC

Most studies about the relationship between gender and medical care needs for HHC service is inconclusive. Studies have shown women are more likely to receive HHC than males, and this gender gap increases by age [8]. Women had higher likelihoods of being hospitalized, contacting ambulatory mental health care, and using over the counter medication than men [9–11]. Yet, men presented with higher levels of need for HHC since men had higher rates of most chronic conditions, limitations in the ADL, and IADL [10]. Admittedly, women are more likely to report functional limitations. However, in the HHC services, men have higher frequencies of ~~using~~ medical care utilization than women, in particular among disable patients [12].

Socioeconomic status and medical care needs

The literature confirms that poverty and level of urbanization are linked with medical care needs [13]. In general, patients with lower socioeconomic status have a lower level of medical care utilization but higher unmet needs in HHC than those with higher socioeconomic status. For instance, Saeed et al. [1] found that patients with lower incomes and living in rural area have less medical care use due to the difficulty of financial accessibility. Forbes et al. [11] also found that patients with lower socioeconomic status had more unmet home care needs than those with higher socioeconomic status since support services of HHC often are expensive, especially for people with dementia.

However, others found that socioeconomic status is not related to medical care needs. For example, Freedman et al. [5] argued that Medicare managed care enrollees with low socioeconomic status have higher accesses to HHC visits. Still, the poorer minority like African American have a greater resource utilization and greater reimbursement of home health services with increased risk for morbidity than the richer white [14]. Thus, whether the lower socioeconomic status is associated with medical care needs remaining uncertain.

Theory and arguments

Guided by the perspective of “fundamental causes of health and disease”, the poorer socioeconomic status is the fundamental causes of disease. Since 1995, Bruce Link and Jo Phelan have confirmed that social conditions cause illness through two pathways [15]. One is those with low socioeconomic status are less likely to have access to health care [15]. The other is that the mechanisms of risky health behaviors such as poor diet, stressful life, heavy smoking, less social support, and higher job hazards could lead more anxiety, depression, higher cholesterol and blood pressure, heart disease [15]. According to the perspective of the feminization of poverty, the inequality in living standards between men and women is increasing at old age, and the majority of old women face the highest risk of poverty due to

lack of income and resources [16]. Based on the theories of fundamental causes of disease and feminization of poverty, we argued that women, patients with lower income, and those who live in more rural areas may be more likely to have a higher level of medical care needs. And female with higher income is less likely to experience a higher level of medical care needs. Hypotheses are as follows:

(1) Women are more likely to have higher levels of medical care needs in HHC than men.
(2) Lower income is more likely to be associated with higher levels of medical care needs in HHC compared to the higher income group.
(3) Women who are the poorer are more likely to associated with higher levels of medical care needs in HHC than those who are the rich.

Material and methods

Data sources and samples

The data for this cross-sectional population-based study was obtained from the National Health Insurance Research Database (NHIRD) from 1997 to 2013 in Taiwan. NHIRD is a nationwide database which contains the data of more than 23 million individuals and is managed by the National Health Research Institute (NHRI) before 2015. National Health Insurance (NHI) system, which is a universal healthcare scheme with single-payer and covers 99% of the population in Taiwan [17], has been launched in 1995 [18]. We conducted a population-based study to examine the relationship between social determinants, diseases, and medical care needs levels in HHC patients. We included all patients who received HHC and 60-year-old or over. The study has been granted exemption from review by the Institutional Review Board of Chi Mei Medical Center (IRB No. 10410-E01).

Outcome variables and covariates

The medical care needs of patients who received HHC in Taiwan is classified by resource utilization groups (RUGs) from level 1 to level 4, and HHC services at different levels of RUGs are all reimbursed with correlated payments by NHI. The first level RUG is intended for those who only require ordinary healthcare visits to home and level 2 to 4 of RUG are aimed at people who require one, two, three or more specialized care services respectively. specialized care services include “change tracheostomy set”, “urinal indwelling catheterization”, “insertion of nasogastric tube”, “bladder irrigation”, “~~would treatment~~”, “IV drip”, “colostomy irrigation”, and so on. Medical care need is an ordinal variable.

We consider three types of covariates including predisposing factors (age and gender), enabling variables (income and urbanization), need factors (major diseases, and comorbidity). Predisposing factors are socio-demographic characteristics: gender and age. We grouped age every 5 years into nine groups from 60-105 years old. We then created 8 gender-income groups were recorded by four income levels and two gender groups.

The enabling variables reflect the location (status) of the individuals in their society as measured by income, education, and urban levels of residence. One of the enabling variables is income, regarded as four levels: one is dependent group, coding as 0, who are usually wives, children, parents and their health insurance covered by their spouses or relatives. The other three levels of income were defined by salary-based health insurance monthly premiums, coding by 1-3: (1) low socioeconomic status (SES): lower than US$571 per month (New Taiwan Dollar (NT$) 20000); (2) moderate SES: between US$571–1141 per month (NT$20000–40000); and (3) high SES: US$1142 per month (NT$40001) or more. This study regarded income as one proxy of individual SES.

The other enabling variables was urbanization level of residence. The urbanization level of residence was classified into four levels of residence from urban to rural areas based on 5 indices in Taiwan: population density, percentage of residents with college-level or higher education, percentage of residents > 65 years old, percentage of residents who are agriculture workers, and the number of physicians per 100000 patients [19].

The need factors include major diseases and comorbidity. The major disease factors are cancer, dementia, cerebrovascular disease, heart failure, chronic obstructive pulmonary disease, chronic liver disease and cirrhosis, and chronic kidney disease. The comorbidity factors are chronic disease consisting of hypertension, diabetes, coronary artery disease, hyperlipidemia, atrial fibrillation, tuberculosis, and epilepsy, defined by International Classification of Diseases, Ninth Revision, Clinical Modification (ICD-9-CM).

Statistical analysis

The dependent variable is the four-level medical care needs, which is one ordinal variable. Firstly, we used χ^2 analysis to examine the differences between each categorical independent variable and the dependent variable. Secondly, we used χ^2 to investigate the differences in medical care needs by eight aged-income groups. Thirdly, we applied multiple-ordinal regression models to examine the relationships between the predisposing factors, need factors, care factors, and medical care needs. All the analysis was conducted by STATA 13 and incorporated the weighted procedure used in NHIRD sampling design.

In the multiple-ordinal regression models, we adopt the statistical method of mediation and moderation. When we examine how one variable affects the other, the next set of research questions to address are when they are related. Statistical techniques testing moderation are among the most widely used data analysis techniques in a variety of disciplines.

Results

Change of HHC patients over time

As shown in Fig 1, there are more and more HHC patients who have been in the second level of RUG from 1997 to 2013. The number of patients within the third level of RUG increased from 1997 to 2008, but then decreased since 2011. More importantly, although the population of HHC patients at the fourth level of RUG has got higher steadily from 1997 to 2005, it started to decline since 2005.

Fig 1 The change of the proportion of medical care need levels during 1997-2013

Characteristics of HHC patients

Table 1 showed patient characteristics. The frequency (percentages) for four levels of medical care need among HHC patients is 1.89%, 49.03%, 41.02%, and 8.07% respectively. The majority of patients are with the first and second levels of income: “the dependent group” (47.61%) and “low” (33.71%). Most respondents’ residences in very urban or urban areas. Among all HHC patients, 16.91% had cancer, 31.29% got dementia, 70.15% had cerebrovascular disease, 25.59% had heart failure, 42.78% got a chronic obstructive pulmonary disease, 19.28% had chronic liver disease and cirrhosis, and 25.59% of the population had chronic kidney disease. These HHC patients have the highest prevalence of hypertension (81.03%) and the lowest prevalence of epilepsy (6.37%).

Table 1. Characteristics for home healthcare patients in Taiwan from 1997 to 2013

Variables	Category	Frequency	Distribution (%)
**Medical Care Needs**	RUG 1	4,495	1.89
	RUG 2	116,839	49.03
	RUG 3	97,756	41.02
	RUG 4	19,230	8.07
**Gender**	Male	116,691	48.96
	Female	121,629	51.04
**age**	60~105 [a]	79.47 [b]	8.37 [b]
**Income** [c]	Dependent	113,468	47.61
	Low	80,329	33.71
	Moderate	43,213	18.13
	High	1,310	0.55
**Urbanization**	Urban	122,484	51.39
	Sub-urban	91,107	38.23
	Sub-rural	21,040	8.83
	Rural	3,689	1.55
**Cancer**	Yes	40,294	16.91
**Dementia**	Yes	74,578	31.29
**Cerebrovascular**	Yes	167,183	70.15
**Heart failure**	Yes	60,983	25.59
**Chronic obstructive pulmonary disease**	Yes	101,947	42.78
**Liver disease**	Yes	45,957	19.28
**Kidney disease**	Yes	60,983	25.59
**Hypertension**	Yes	193,116	81.03
**Diabetes**	Yes	27,467	11.53
**Coronary artery disease**	Yes	108,160	45.38
**Hyperlipidemia**	Yes	72,847	30.57
**Atrial fibrillation**	Yes	32,903	13.81
**Tuberculosis**	Yes	16,410	6.89
**Epilepsy**	Yes	15,177	6.37

The datasets only include home health care respondents and total number of cases is 238,320.
[a] Age range is from age 60 to age 105.
[b] The mean (standard deviation) are shown for the variables: age.
[c] Income dependent group refers to people whose health insurance of premium were covered by their family members who have income. And the other three income levels were defined by salary-based health insurance premiums.

Factors related to medical care need

Differences in medical care need at the baseline among HHC patients is shown in Table 2. The percentages of females are increasing from RUG 1 to RUG 4. 54.02% and 35.21% of the population with the income level at "the dependent group" and "low" are in the fourth level of RUG. There are 51.69% of patients who lived in a very urban area.

Table 2 presents the largest group with major diseases in each level of RUG compared to the counterpart without the major diseases. HHC patients with cerebrovascular disease are more likely to have a higher level of RUG compared to patients without cerebrovascular diseases. HHC patients with kidney diseases are significantly less likely to have a high level of RUG compared to patients without kidney diseases.

Table 2. Distribution in age-specific, gender-specific, socioeconomic-specific, disease-specific estimates for medical care need levels

	RUG 1	RUG 2	RUG 3	RGU 4
**Male**	51.52	51.27	45.93	49.77
**Female**	48.48	48.73	54.07	50.23
**Income dependent**	45.90	45.47	48.99	54.02
**Low income** [a]	35.93	33.83	33.16	35.21
**Moderate income** [a]	16.84	20.11	17.41	10.09
**High income** [a]	1.33	0.60	0.43	0.68
**Urbanization 1 (urban)**	52.12	51.65	51.00	51.69
**Urbanization 2 (sub-urban)**	38.87	38.10	38.31	38.40
**Urbanization 3 (sub-rural)**	7.63	8.70	9.09	8.52
**Urbanization 4 (Rural)**	1.38	1.54	1.60	1.39
**Cancer**	22.20	19.08	14.93	16.91
**Dementia**	18.64	32.27	31.82	25.60
**Cerebrovascular**	50.90	67.94	72.65	75.41
**Heart failure**	33.79	26.80	24.63	21.20
**Chronic obstructive pulmonary disease**	49.79	43.59	41.79	41.19
**Liver disease**	20.82	20.36	18.59	15.90
**Chronic Kidney disease**	18.40	19.85	17.32	13.98
**Hypertension**	75.39	81.85	81.04	77.32
**Diabetes**	10.75	11.26	11.93	11.22
**Coronary artery disease**	45.98	47.04	44.65	38.92
**Hyperlipidemia**	31.08	32.00	30.15	23.88
**Atrial fibrillation**	15.35	14.20	13.51	12.58
**Tuberculosis**	10.50	7.32	6.29	6.41
**Epilepsy**	4.05	5.98	6.61	8.03

Note: The datasets only include home health care respondents and the total number of cases is 238,320. The numbers in the table show the percentage within each level of medical care needs, representing by RUGs, stratified by each disease. All indicates the significance $p < 0.001$ by $\chi 2$ tests.

[a] Income dependent group refers to people whose health insurance of premium were covered by their family members who have income. And the other three income levels were defined by salary-based health insurance premiums.

Multiple ordered-logistic regression models for medical care needs in HHC

In Table 3, we found men are less likely to associated with higher levels of medical care needs than women. Also, a greater age is significantly associated with lower levels of medical care needs. After controlling income and urbanization, women have a 1.20 times higher probability of having medical care needs than men based on Model 2. Those with income level 1, 2, 3 has 3%, 23%, and 26% lower odds of having higher medical care needs compared to those with Income level 0, which is the income-dependent group. Those who live in the sub-urban, sub-rural, and rural areas have 1.05, 1.11, 1.09 times higher odds of having higher medical care needs than those living in the urban areas according to Model 2.

All major diseases are associated with lower levels of medical care needs but the cerebrovascular disease is an exception. HHC patients with cerebrovascular diseases are 1.38 times greater chance to have a higher level of medical care needs compared to those with other major diseases. Furthermore, we found socioeconomic status can only mediate the effect of cerebrovascular disease on higher levels of medical care needs. More importantly, Model 3 showed women with the highest income level have fewer odds of having medical care needs than women with income dependent group for 31%, indicating moderation effects of gender and income on medical care needs are significantly large.

Table 3. Estimated odds for having medical care needs in home healthcare under the impacts of mediators and moderators

	Model 1	Model 2	Model 3
**Predisposing factors**			
**Gender**	1.23*** [1.21,1.25]	1.23*** [1.21,1.25]	1.24*** [1.21,1.27]
**Age** [a]	0.99*** [0.98,0.99]	0.99*** [0.99,0.99]	0.99*** [0.98,0.99]
**Enabling factors**			
**Low income** [b]		0.97*** [0.95,0.98]	0.98 [0.96,0.997]
**Moderate income** [b]		0.77*** [0.75,0.79]	0.79*** [0.76,0.81]
**High income** [b]		0.74*** [0.66,0.82]	0.75*** [0.67,0.84]
**Rurality (Sub-urban)** [c]		1.05*** [1.04,1.07]	1.05*** [1.04,1.07]
**Rurality (Sub-rural)** [c]		1.11*** [1.07,1.14]	1.11*** [1.07,1.14]
**Rurality (Rural)** [c]		1.09* [1.02,1.16]	1.09* [1.02,1.16]
**Major diseases**			
**Cancer**	0.76*** [0.75,0.780]	0.76*** [0.75,0.78]	0.76*** [0.745,0.778]
**Dementia**	0.96*** [0.95,0.98]	0.96*** [0.95,0.98]	0.96*** [0.947,0.979]
**Cerebrovascular**	1.39*** [1.36,1.41]	1.38*** [1.35,1.40]	1.38*** [1.35,1.40]
**Chronic Kidney Disease**	0.89*** [0.86,0.91]	0.89*** [0.87,0.91]	0.89*** [0.87,0.91]
**Chronic obstructive pulmonary disease**	1.02*** [1.01,1.04]	1.02*** [1.01,1.04]	1.02*** [1.01,1.04]
**Liver disease**	0.91*** [0.89,0.93]	0.92*** [0.90,0.94]	0.92*** [0.90,0.94]
**Heart failure**	0.91*** [0.90,0.93]	0.91*** [0.90,0.93]	0.91*** [0.90,0.93]
**Comorbidities**			
**Hypertension**	0.90*** [0.89,0.92]	0.91*** [0.89,0.93]	0.91*** [0.89,0.93]
**Diabetes**	1.07*** [1.04,1.10]	1.07*** [1.04,1.09]	1.07*** [1.04,1.09]
**Coronary artery**	0.93*** [0.92,0.95]	0.93*** [0.92,0.95]	0.94*** [0.92,0.95]
**Hyperlipidemia**	0.84*** [0.82,0.85]	0.85*** [0.83,0.86]	0.84*** [0.83,0.86]
**Atrial fibrillation**	0.97*** [0.95,0.99]	0.97*** [0.95,0.99]	0.97*** [0.95,0.99]
**Tuberculosis**	0.92*** [0.89,0.95]	0.92*** [0.89,0.95]	0.92*** [0.89,0.95]

**Epilepsy**	1.12*** [1.08,1.14]	1.12*** [1.08,1.15]	1.12*** [1.08,1.15]
**Interaction terms** [d]			
**Gender*Low income**			0.97 [0.94,1.00]
**Gender*Moderate income**			0.98 [0.94,1.02]
**Gender*High income**			0.69** [0.53,0.89]

Note: The datasets only include home health care respondents and the number of observation is 238,320. The odds ratios and confidence interval for each medical care need level, representing by RUGs, are shown.

[a] Age range is from age 60 to age 105.

*** indicates p<0.001, *** indicates p<0.01,**indicates p<0.05

[b] Income variable's reference group is level 0 (income dependent)

[c] Rurality variable's reference group is urban area

[d] Interaction term variable's reference group is female with income level 0 (income dependent).

Gender and income related to medical care need

Table 4 and Fig 2 display the association between gender-income group and medical care need levels. We found out there is a significant difference in percentages of RUG levels among eight -income groups. Overall, HHC female patients with low income and moderate income are more likely to have high levels of medical care need. For example, the percentage of HHC female patients with poorer income is 26.43%, 26.24%, 30.68%, 32.07% from RUG 1to RUG 4 respectively compared to other gender-income groups. ~~However,~~ the percentage of females with high income are lower in RUG 1 to 4 compared to males with high income. In particular, cancer patients experienced larger gender gaps in each RUG category at the same level of income.

Table 4. The relationships between sex, income and medical care need level (RUGs) in home healthcare

	RUG1 All	RUG1 Cancer	RUG2 All	RUG2 Cancer	RUG3 All	RUG3 Cancer	RUG4 All	RUG4 Cancer
**Male & Income dependent group (%)**	19.47	21.19	19.23	20.13	18.31	19.57	21.94	23.54
**Female & Income dependent group (%)**	26.43	22.44	26.24	22.45	30.68	25.12	32.07	25.66
**Male & Low income (%)**	22.60	25.70	22.20	25.17	20.07	23.50	22.15	25.61
**Female & Low income (%)**	13.33	12.08	11.63	10.03	13.09	11.27	13.05	11.49
**Male & Moderate income (%)**	8.50	10.26	9.38	11.30	7.22	9.60	5.14	7.29
**Female & Moderate income (%)**	8.34	6.23	10.73	9.95	10.19	10.22	4.96	5.43
**Male & High income (%)**	0.96	1.44	0.46	0.78	0.33	0.57	0.54	0.85
**Female & High income (%)**	0.38	0.67	0.14	0.17	0.10	0.14	0.15	0.13

Note: 1. The total number of cases is 238,320.
2. The results for $\chi 2$ tests with p values below 0.001 are shown.
3. The numbers show the percentage within each level of Medical Care Need, representing by RUG, stratified by each gender-income group.
4. Income dependent group refers to people whose health insurance of premium were covered by their family members who have income. And the other three income levels were defined by salary-based health insurance premiums.

Fig 2 The Gender, Income and Distribution of Medical Care Need Levels

Note: 1. Rug distribution in each gender-income group are significantly different (p-value < 0.001 by $\chi 2$ test).
2. The numbers show the percentage within each level of Medical Care Need Level stratified by each gender-income group for A. whole sample, B. cancer patients, and stroke patients.
3. Income dependent group refers to people whose health insurance of premium were covered by their family members who have income. And the other three income levels were defined by salary-based health insurance premiums.
4. Blue, orange, gray, and yellow represented RUG1~4 respectively.

Discussion

Few studies can display the trend of medical care needs over time [21–24]. We utilized the NHIRD in Taiwan from 1997 to 2013 to scrutinize the pattern of medical care needs. We found that women, the low income, and the poor women have a higher likelihood of having medical care needs than men, the rich, and the rich women respectively. Besides, income and urbanization can mediate and moderate the effect of gender on medical care needs. According to fundamental causes of health and disease and feminization of poverty, being a female and having lower socioeconomic status become the double burden of diseases.

In general, there were increasing frequencies of the first level and the second level of RUG but the frequency of the most severe level of RUG was declining. The trends can be explained by the dynamic equilibrium of morbidity hypothesis, suggesting the population who suffer from disease or disability with less severe conditions are increasing due to the longevity [21–25]. But the most morbidity in life was squeezing into a shorter period of past decades [25–29].

Despite several medical and social elucidations that have been articulated in attempts to account for the differences of medical care needs for HHC, how gender and income ~~underlying~~ this association remains unclear, especially for the older population [8,12]. In our study, we found significant gender and income gaps in medical care needs. In particular, women and the poorer are more likely to associated with a higher level of medical care needs than men and the rich respectively. Moreover, women with higher income have lower odds of medical care needs than men and women with a poorer income, corroborating Hypothesis 3. Least but not last, disease patterns are significantly related to medical care needs but showed in different ways. In particular, cerebrovascular disease, diabetes, and epilepsy are associated with higher levels of RUG. But other major diseases such as cancer, dementia, heart failure, chronic liver disease and cirrhosis, and chronic kidney disease and other comorbidity factors

including hypertension, coronary artery disease, hyperlipidemia, atrial fibrillation, and tuberculosis have negative links with medical care needs in HHC.

Consistent with prior results, females have higher medical care need [8,9,11,30]. Our finding also suggested that there are gender differences in dissimilar levels of medical care needs [31]. An increasing proportion of women was noted in the third and fourth level of RUG. It could be because women suffer much more from nonlethal illness and also more lethal illness than men for several conditions [32], have a higher degree of morbidity in old age than male on average, especially in a higher rate of chronic debilitation disorders [33]. In particular, women in the income-dependent group have much higher frequencies of increased medical care needs than women in other income groups, while men in the poor income group have increased medical care needs but the proportion is similar to men in the income-dependent group. The findings confirmed the theory of feminization of poverty [16].

Moreover, we also agree that cerebrovascular disease has the highest probability of increased medical care needs for HHC patients[8]. And epilepsy, diabetes, and chronic obstructive pulmonary disease rank after. Remarkably, those who have diabetes utilized higher level of RUG can moderately be explained by lower-income and living in rural areas. This situation is the case of fundamental causes of health and disease, suggesting worse social condition make patients unhealthy and have higher levels of RUG [15].

Limitations and strengths

Due to the cross-sectional design, the study cannot reveal any causal inferences but only the associations between social determinants and medical care need. Second, since the NHIRD is claim-based routinely collected dataset, it does not provide some specific socioeconomic data such as education years, out-of-pocket expense for healthcare, or other economic circumstances. Third, the evaluation of medical care needs was based on the need for special

medical care items and on the time-point when patients received HHC for the first time. The change in medical care needs cannot be identified in our study.

Conclusion

The findings fitted dynamic equilibrium of morbidity hypothesis, put forward by Manton, view mortality reductions as the result of decreases in the rate of chronic disease progression [25–27], so the severe levels of medical care need are reducing whereas the basic levels of medical care need for HHC are climbing over time [34–36]. In addition, the results correspond with previous research, which indicates that lower socioeconomic status can partially explain the higher level of medical care need among women. The results are consistent with the theories of fundamental causes of disease and feminization of poverty. To reduce the effect of poverty on women's higher medical care need, policymakers could provide financial supports and more specific medical resources for the female elderly in the income-dependent or low-income groups, especially for HHC patients.

References

1. Saeed BII, Yawson AE, Nguah S, Agyei-Baffour P, Emmanuel N, Ayesu E. Effect of socio-economic factors in utilization of different healthcare services among older adult men and women in Ghana. BMC Health Serv Res [Internet]. 2016 Aug 16 [cited 2019 Dec 11];16. Available from: https://www.ncbi.nlm.nih.gov/pmc/articles/PMC4987977/
2. West JA, Miller NH, Parker KM, Senneca D, Ghandour G, Clark M, et al. A Comprehensive Management System for Heart Failure Improves Clinical Outcomes and Reduces Medical Resource Utilization. Am J Cardiol. 1997 Jan 1;79(1):58–63.
3. DeVries A, Li C-HW, Sridhar G, Hummel JR, Breidbart S, Barron JJ. Impact of medical homes on quality, healthcare utilization, and costs. - Abstract - Europe PMC. Am J Manag Care. 2012;18(9):534–44.
4. Sheets L, Popejoy L, APRN G-B, Khalilia M, Petroski G, Parker JC. Identifying Patients at Risk of High Healthcare Utilization. AMIA Annu Symp Proc. 2017 Feb 10;2016:1129–38.
5. Freedman VA, Rogowski J, Wickstrom SL, Adams J, Marainen J, Escarce JJ. Socioeconomic Disparities in the Use of Home Health Services in a Medicare Managed Care Population. Health Serv Res. 2004 Oct;39(5):1277–98.
6. Stewart S, Pearson S, Luke CG, Horowitz JD. Effects of home-based intervention on unplanned readmissions and out-of-hospital deaths. J Am Geriatr Soc. 1998 Feb;46(2):174–80.
7. Anderson C, Rubenach S, Mhurchu CN, Clark M, Spencer C, Winsor A. Home or hospital for stroke rehabilitation? results of a randomized controlled trial : I: health outcomes at 6 months. Stroke. 2000 May;31(5):1024–31.
8. Chang H-T, Lai H-Y, Hwang I-H, Ho M-M, Hwang S-J. Home healthcare services in Taiwan: a nationwide study among the older population. BMC Health Serv Res. 2010

Sep 21;10(1):274.

9. Gerritsen AA, Devillé WL. Gender differences in health and health care utilisation in various ethnic groups in the Netherlands: a cross-sectional study. BMC Public Health. 2009 Apr 20;9(1):109.

10. Gruneir A, Forrester J, Camacho X, Gill SS, Bronskill SE. Gender differences in home care clients and admission to long-term care in Ontario, Canada: a population-based retrospective cohort study. BMC Geriatr. 2013 May 16;13:48.

11. Forbes DA, Jansen SL, Markle-Reid M, Hawranik P, Morgan D, Henderson S, et al. Gender differences in use and availability of home and community-based services for people with dementia. Can J Nurs Res Rev Can Rech En Sci Infirm. 2008 Mar;40(1):39–59.

12. Lee Y-H, Lu C-W, Huang C-T, Chang H-H, Yang K-C, Kuo C-S, et al. Impact of a home health care program for disabled patients in Taiwan: A nationwide population-based cohort study. Medicine (Baltimore). 2019 Feb;98(7):e14502.

13. National Academies of Sciences, Engineering, and Medicine, Health and Medicine Division, Board on Health Care Services, Committee on Health Care Utilization and Adults with Disabilities. Health-Care Utilization as a Proxy in Disability Determination [Internet]. Washington (DC): National Academies Press (US); 2018 [cited 2019 Dec 11]. Available from: http://www.ncbi.nlm.nih.gov/books/NBK500102/

14. Chin MH, Zhang JX, Merrell K. Diabetes in the African-American Medicare Population: Morbidity, quality of care, and resource utilization. Diabetes Care. 1998 Jul 1;21(7):1090–5.

15. Link BG, Phelan J. Social Conditions As Fundamental Causes of Disease. J Health Soc Behav. 1995;80–94.

16. Stone RI. The Feminization of Poverty among the Elderly. Womens Stud Q.

1989;17(1/2):20–34.
17. Lee Y-H, Ang TFA, Chiang TC, Kaplan WA. Growing concerns and controversies to Taiwan's National Health Insurance—what are the lessons from mainland China, South Korea and Singapore? Int J Health Plann Manage. 2018;33(1):e357–66.
18. Liang L-L. Impact of integrated healthcare: Taiwan's Family Doctor Plan. Health Policy Plan. 2019 Nov 1;34(Supplement_2):ii56–66.
19. Liu C-Y, Hung Y-T, Chuang Y-L, Chen Y-J, Weng W-S, Liu J-S, et al. Incorporating Development Stratification of Taiwan Townships into Sampling Design of Large Scale Health Interview Survey. J Health Manag. 2006;4(1).
20. Fries JF. Aging, natural death, and the compression of morbidity. N Engl J Med. 1980 Jul 17;303(3):130–5.
21. Manton K, Gu X. Changes in the Prevalence of Chronic Disability in the United States Black and Nonblack Population Above Age 65 from 1982 to 1999. Proc Natl Acad Sci U S A. 2001 Jun 1;98:6354–9.
22. Crimmins E, Saito Y. Change in the Prevalence of Diseases Among Older Americans: 1984-1994. Demogr Res [Internet]. 2000 Nov 1 [cited 2020 Feb 15];3(9). Available from: https://www.demographic-research.org/volumes/vol3/9/
23. Schoeni R, Freedman V, Martin L. Why Is Late-Life Disability Declining? Milbank Q. 2008 Apr 1;86:47–89.
24. Fries JF. Aging, natural death, and the compression of morbidity. N Engl J Med. 1980 Jul 17;303(3):130–5.
25. Cai L, Lubitz J. Was there compression of disability for older Americans from 1992 to 2003? Demography. 2007 Aug 1;44(3):479–95.
26. Sundberg L, Agahi N, Fritzell J, Fors S. Trends in health expectancies among the oldest old in Sweden, 1992-2011. Eur J Public Health. 2016;26(6):1069–74.

27. Crimmins EM. Trends in the health of the elderly. Annu Rev Public Health. 2004;25:79–98.
28. Crimmins EM, Zhang Y, Saito Y. Trends Over 4 Decades in Disability-Free Life Expectancy in the United States. Am J Public Health. 2016 Jul;106(7):1287–93.
29. Freedman VA, Martin LG, Schoeni RF. Recent trends in disability and functioning among older adults in the United States: a systematic review. JAMA. 2002 Dec 25;288(24):3137–46.
30. Sørbye LW, Finne-Soveri H, Ljunggren G, Topinková E, Bernabei R. Indwelling catheter use in home care: elderly, aged 65+, in 11 different countries in Europe. Age Ageing. 2005 Jul;34(4):377–81.
31. Vegda K, Nie JX, Wang L, Tracy CS, Moineddin R, Upshur RE. Trends in health services utilization, medication use, and health conditions among older adults: a 2-year retrospective chart review in a primary care practice. BMC Health Serv Res. 2009 Nov 30;9(1):217.
32. Siegel JS. The Demography and Epidemiology of Human Health and Aging [Internet]. Springer Netherlands; 2012 [cited 2020 Oct 15]. Available from: https://www.springer.com/us/book/9789400713147
33. Rieker PP, Bird CE. Rethinking Gender Differences in Health: Why We Need to Integrate Social and Biological Perspectives. J Gerontol B Psychol Sci Soc Sci. 2005 Oct 1;60(Special Issue 2):S40–7.
34. Graham P, Blakely T, Davis P, Sporle A, Pearce N. Compression, expansion, or dynamic equilibrium? The evolution of health expectancy in New Zealand. J Epidemiol Community Health. 2004 Aug;58(8):659–66.
35. Manton KG. Changing concepts of morbidity and mortality in the elderly population. Milbank Mem Fund Q Health Soc. 1982;60(2):183–244.

36. Hossin MZ, Östergren O, Fors S. Is the Association Between Late Life Morbidity and Disability Attenuated Over Time? Exploring the Dynamic Equilibrium of Morbidity Hypothesis. J Gerontol B Psychol Sci Soc Sci. 2019 Oct 4;74(8):e97–106.

Figure

Click here to access/download;Figure;Fig1.tiff

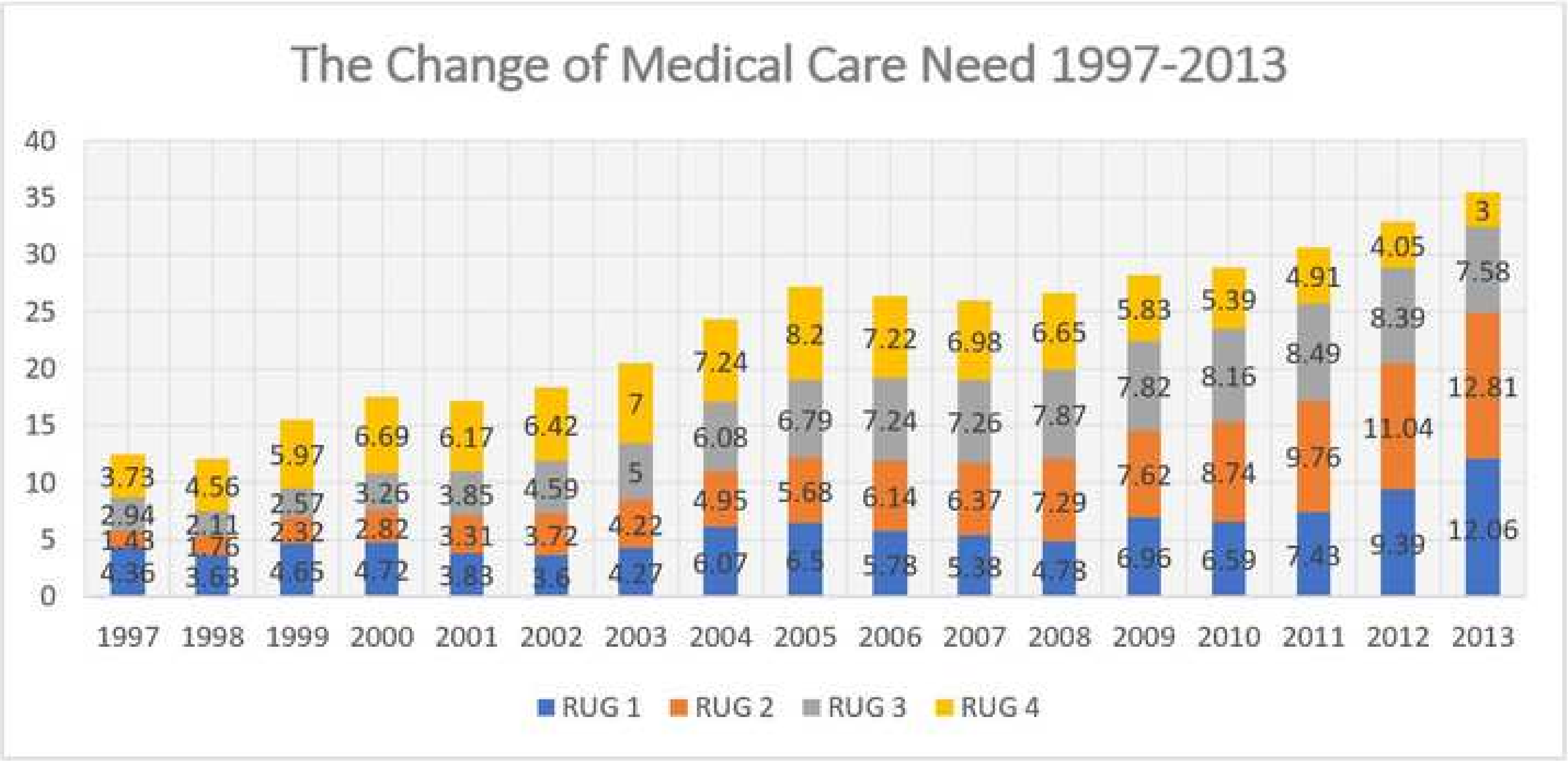

Figure
Click here to access/download;Figure;Fig 2.tif

A. For All Patients

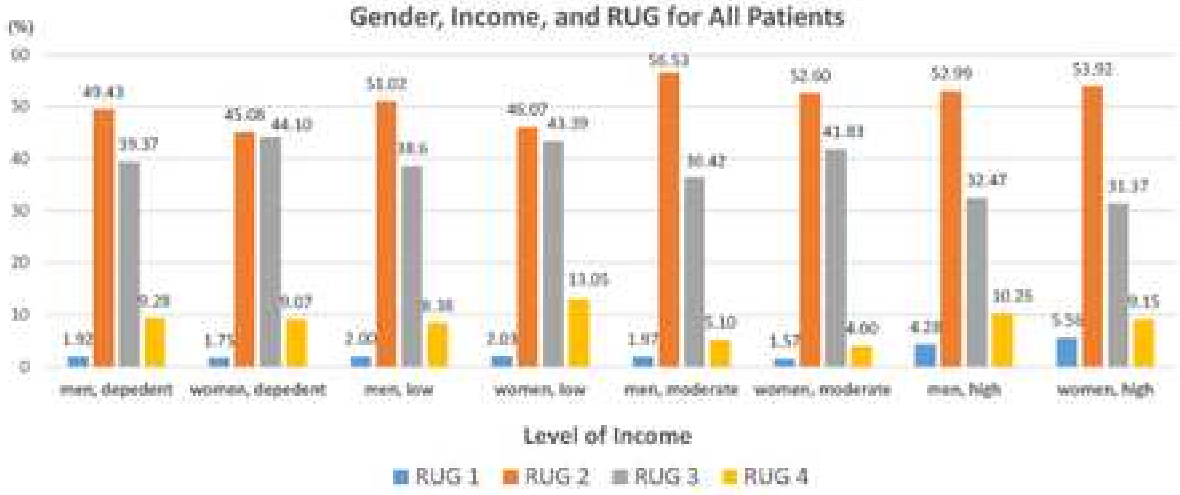

B. For Cancer Patients

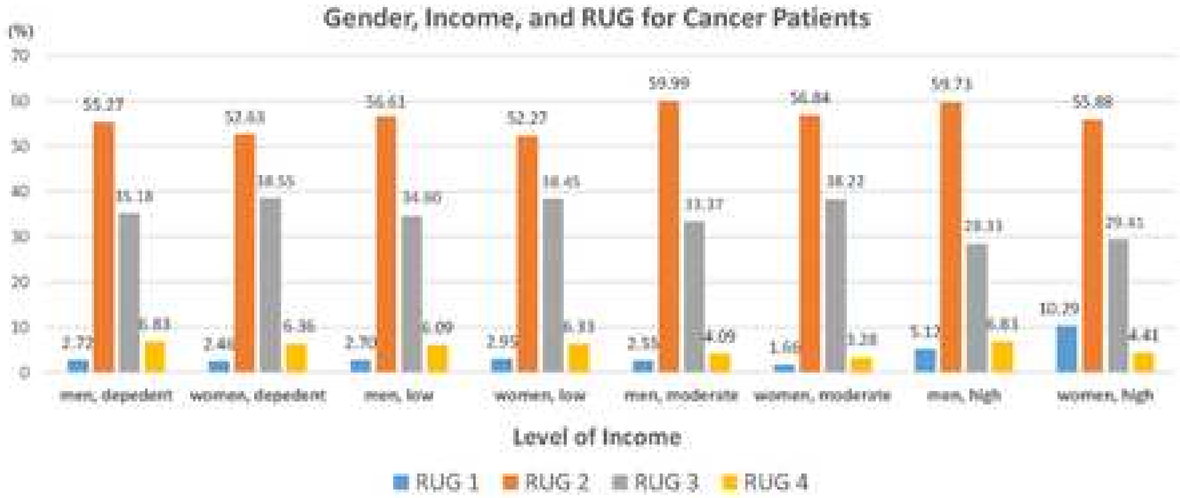

C. For Stroke Patients

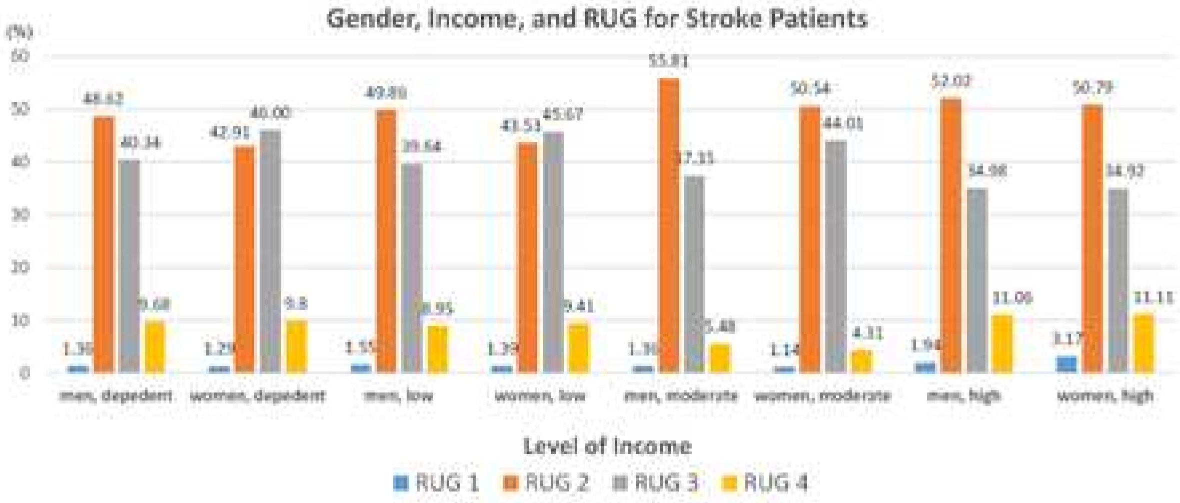

Supplement: Attachment — Submitted filename: PONE-D-20-32788_TZB_comments.pdf [file pone.0247622.s001.pdf]
